# TRIM24介导肺癌细胞吉非替尼耐药机制探讨

**DOI:** 10.3779/j.issn.1009-3419.2016.01.03

**Published:** 2016-01-20

**Authors:** 海英 李, 庆苓 王, 海军 包, 恒 张, 莹 庄

**Affiliations:** 221000 徐州，徐州医学院基础医学院病理教研室 Department of Pathology, College of Basic Medical Science, Xuzhou Medical College, Xuzhou 221000, China

**Keywords:** 肺肿瘤, 吉非替尼, 表皮生长因子受体酪氨酸激酶抑制剂, Lung neoplasms, Geftinib, Epidermal growth factor receptor tyrosine kinase inhibitor

## Abstract

**背景与目的:**

表皮生长因子受体酪氨酸激酶抑制剂(epidermal growth factor receptor tyrosine kinase inhibitor, EGFR-TKI)的原发和继发性耐药已成为其在临床肺癌治疗中的拦路虎，在肺癌组织中研究发现TRIM24呈高表达状态，在肺癌细胞中能调控细胞增殖、周期和细胞凋亡，本研究旨在进一步探讨TRIM24调控肺癌细胞吉非替尼耐药的分子机制。

**方法:**

应用MTT方法和流式细胞仪检测干扰TRIM24及干扰TRIM24后加入吉非替尼对肺癌细胞系增殖能力及凋亡率的抑制率的变化，同时应用Western blot方法检测凋亡相关基因的变化及AKT信号通路中蛋白的表达。

**结果:**

在A549细胞中使用干扰TRIM24可以提高吉非替尼对肿瘤增殖能力的抑制率，增加吉非替尼诱导的凋亡水平，同时干扰TRIM24及干扰TRIM24加入吉非替尼后促凋亡相关基因p-BAD、Bcl-2和AKT及AKT信号通路相关蛋白PIK3CA均出现降低。

**结论:**

TRIM24能够调控肺癌细胞吉非替尼耐药，并且通过AKT信号通路引起肺癌细胞EGFRTKI耐药。

## Abstract (en)

**Background and objective:**

Epidermal growth factor receptor tyrosine kinase inhibitor (EGFR-TKI) resistance signifcantly limits its use in clinical practice. Study found that TRIM24 was overexpressed in non-small cell lung cancer (NSCLC) tissues and regulate cell growth, cell cycle and apoptosis in lung cell lines. Te aim of this study is to explore the mechanism of TRIM24 to regulate resistance of Geftinib in NSCLC cells.

**Methods:**

MTT and apoptosis were used to detect the change of cell grow and cell apoptosis with down-expression TRIM24 and ShTRIM24 with presence of Geftinib. Meanwhile, Western blot was used to detect the expression of protein related to apoptosis and AKT signal path.

**Results:**

TRIM24 interference could improve the effect of gefitinib on cell growth inhibition and upregulate the cell apoptosis in A549 cell. Down-regulated of endogenous TRIM24 and ShTRIM24 with Giftinib could also reduce the protein related apoptosis, such as p-BAD and Bcl-2, and the protein PIK3CA related AKT signal path in A549 cell.

**Conclusion:**

TRIM24 could regulate required resistance to Geftinib via Akt pathway in NSCLC.

在过去的30年里我国肺癌的发生率逐步上升，与其他国家一样其死亡率已经居恶性肿瘤之首，手术是治愈非小细胞肺癌(non-small cell lung cancer, NSCLC)的有效方法，但只有约30%的NSCLC患者拥有手术机会，而放化疗治疗NSCLC的疾病缓解率分别只有25%-35%和15%-20% ^[1]^。近年来以表皮生长因子受体酪氨酸激酶抑制剂(epidermal growth factor receptor tyrosine kinase inhibitor, EGFR-TKI)为靶点的肿瘤分子靶向治疗的研究成为了肿瘤治疗研究的热点，其中以吉非替尼和厄洛替尼为代表^[2]^，然而大多数患者并不能从这一疗法中获益，究其原因是这些患者最终都表现为对抗EGFR疗法产生耐药，包括原发性和继发性耐药。因此，探究提高EGFR靶向治疗敏感性的分子机制以及干预耐药的靶点在NSCLC的临床靶向治疗中具有重要的临床意义。

TRIM24是三重基序蛋白家族中的一员，最初命名为转录调节因子1α(transcription intermediary factor 1α, TIF1α)，是维甲酸信号通路的共调节因子^[3-5]^。TRIM24异常表达可能通过多种分子机制促进肿瘤的发生发展。早期研究^[6-8]^证实TRIM24通过染色体异位形成致癌性融合蛋白从而促进急性早幼粒细胞白血病、乳头状甲状腺癌及骨髓增生异常综合征等肿瘤的发生发展。TRIM24在正常的胚胎干细胞中存在大量的细胞核表达状态，随着分化和器官形成而逐渐减少，仅仅在少数器官中残存如生殖器^[9, 10]^，最近研究证实从乳腺导管上皮细胞到乳腺浸润性导管癌中TRIM24的表达逐渐增多，且随着TRIM24表达的增加，临床预后越差^[11]^，TRIM24能促进P53泛素化降解，因此可作为一个治疗靶点以恢复P53的肿瘤抑制功能来治疗肿瘤^[12]^。TRIM24能够通过与雌激素受体结合而激活雌激素受体依赖性蛋白从而促进肿瘤细胞的增殖及肿瘤的发展^[13, 14]^。但TRIM24是如何促进肺癌发生发展这一分子机制尚不清楚，TRIM24是否与临床肺癌化疗耐药相关更未见报道。

## 1 材料和方法

### 1.1 主要试剂

人肺癌细胞系A549购自American Type Culture Collection(Manassas, VA, USA)。细胞培养用DMEM高糖培养基购自Gibco，胎牛血清购自碧云天。ShRNA-TRIM24(M-005387-03-0005)和阴性对照ShRNA#1(D-001810-01-20)购自Dharmacon，转染试剂购自Qiagen。RNA提取试剂，反转录试剂盒、Realtime PCR试剂盒及PCR引物合成购自Takara。P-BAD、BAD、Bcl-xL、Bcl-2、p-AKT、AKT、P85、PIK3CA和PTEN单克隆抗体购自CST；TRIM24单克隆抗体购自proteintech；β-actin抗体购自Santa Cruz；辣根过氧化物酶标记的山羊抗小鼠及山羊抗兔IgG购自Santa Cruz。BCA法蛋白定量试剂盒购自碧云天，裂解液及超敏发光试剂盒购自Pierce。流式凋亡检测试剂盒购自BD。

### 1.2 细胞培养

肺癌细胞系A549使用含有10%的小牛血清DMEM培养基，37 ℃、5%CO_2_的条件下培养，每两天换一次液，并用0.25%的胰蛋白酶进行消化传代。取对数生长的细胞，分为三组：乱序对照组(ShCON)、ShTRIM24组和ShTRIM24+Gifitinib组。每次实验同一个处理因素设两个复孔，重复3次实验。

### 1.3 实时定量PCR

细胞提取总R N A后，反转录成cDNA，使用SYBR Green法，进行Real-time PCR扩增，总体积20 μL。扩增过程如下：95 ℃，30 s；95 ℃，5 s；60 ℃，30 s，40个循环。β-actin作为内参。基因相对表达水平计算方式如下：ΔCt=Ct_gene_-Ct_reference_，增加倍数用2^-ΔΔCt^方法计算。每次试验均做3个重复孔。

### 1.4 MTT法检测细胞增殖实验

将单个细胞悬液接种于96孔培养板中，每孔体积200 μL含1, 500个细胞，培养24 h，每孔加入10 μL的CCK8试剂(Dojindo, Gaithersburg, MD)继续培养4 h，取出后450 nm波长下测定各孔光吸收值，以不含细胞的等体积培养基作对照，连续测量5天，根据数值绘制细胞生长曲线。

### 1.5 细胞凋亡检测

细胞分为三组乱序对照组(ShCON)、ShTRIM24组和ShTRIM24+Gifitinib组，使用PBS清洗两次，0.25%胰蛋白酶消化，用培养基终止消化后，将细胞收集到EP管中，1, 000 rpm 4 ℃离心5 min后，去上清。用PBS洗两遍后每个样本中加400 μL缓冲液，吹打成单细胞悬液，后避光加入FITC/Annexin V 10 μL和PI 5 μL染色20 min后上机检测。

### 1.6 Western blot法检测蛋白表达

收集细胞并加入裂解液充分裂解，低温高速离心(4 ℃, 12, 000 rpm/min, 30 min)，提取上清为总蛋白。每个泳道加入总蛋白60 μg，12%SDS-PAGE凝胶电泳，转印(60 V, 120 min)到PVDF上。5%牛血清白蛋白室温封闭2 h。抗P-BAD、BAD、Bcl-xL、Bcl-2、p-AKT、AKT、P85、PIK3CA、PTEN、TRIM24和β-actin(1:1, 000) 4 ℃孵育过夜。分别与对应的二抗(1:2, 000)室温孵育2 h，ECL显色，结果经自动电泳凝胶成像分析仪采集。

### 1.7 统计学方法

采用SPSS 16.0统计软件分析数据，数据用Mean±SD表示，组间比较采用*t*检验，以*P* < 0.05为差异有统计学意义。

## 2 结果

### 2.1 肺癌细胞对EGFR-TKI药物的敏感性与TRIM24的表达水平相关

应用MTT和流式细胞仪检测肺癌细胞系A549中干扰内源性TRIM24及干扰TRIM24后加入吉非替尼后细胞增殖和凋亡的变化，结果显示：在A549细胞中干扰TRIM24后细胞增殖减弱，而干扰TRIM24同时加入吉非替尼(1 μmol/L)后细胞增殖明显减弱(*P* < 0.05)，因此TRIM24可以提高吉非替尼对肿瘤增殖能力的抑制率(图 1A)。在A549细胞中干扰TRIM24后细胞凋亡增加(ShCON *vs* ShTRIM24：2.72±0.32 *vs* 5.27±0.40, *P* < 0.05)，而干扰TRIM24同时加入吉非替尼组凋亡明显增加(ShTRIM24 *vs* ShTRIM24+Giftinib: 5.27 ±0.40 vs 7.19±0.33, *P* < 0.05)，因此TRIM24增加了吉非替尼诱导的凋亡水平(图 1B)。

**1:**
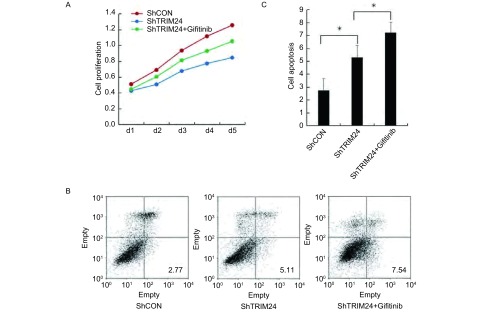
肺癌细胞对EGFR-TKI药物的敏感性与TRIM24的表达水平相关。A：在A549细胞中干扰TRIM24抑制细胞增殖，而干扰TRIM24同时加入吉非替尼（1 *μ*mol/L）后对肿瘤增殖能力抑制更为明显；B、C：凋亡检测结果显示，A549细胞中干扰TRIM24增加了细胞凋亡水平，而干扰TRIM24同时加入吉非替尼诱导的细胞凋亡更为明显。^*^：*P* < 0.05。 The sensitivity of lung cancer cells to EGFR-TKI drug associated with the expression level of TRIM24. A: Tranfection of ShTRIM24 can improve the inhibition of cell proliferation, and ShTRIM24 with Gefitinib can reduce cell proliferation obviously in A549 cell; B, C: Tranfection of ShTRIM24 can improve the cell apoptosis and ShTRIM24 with Gefitinib can improve the cell apoptosis obviously in A549 cell. EGFR-TKI: epidermal growth factor receptor tyrosine kinase inhibitor. ^*^: *P* < 0.05.

### 2.2 TRIM24在肺癌细胞系A549和PC9中对凋亡相关蛋白的影响

在肺癌细胞系A549中干扰TRIM24后收集细胞，提取总蛋白，用Western blot方法检测TRIM24及凋亡相关蛋白P-BAD、BAD、Bcl-xL、Bcl-2等蛋白表达，干扰TRIM24后P-BAD表达明显降低，而BCL-2的表达略微降低，而干扰TRIM24同时加入吉非替尼后P-BAD和BCL-2降低更为明显(图 2)。

**2:**
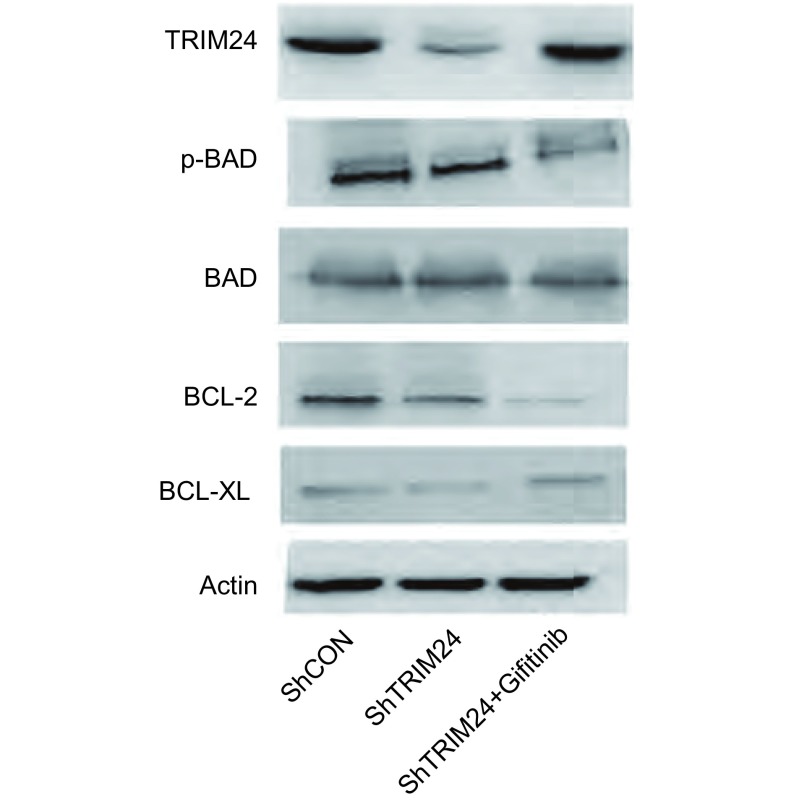
敲除TRIM24及同时加入吉非替尼引起凋亡相关蛋白的变化。敲除肺癌A549细胞中内源性TRIM24后凋亡相关基因*p-BAD*表达下调，BCL-2略微下调，而干扰TRIM24同时加入吉非替尼后凋亡相关基因*p-BAD*、*BCL-2*的表达降低更为明显。 The expression of protein related apoptosis with tranfection of ShTRIM24 and ShTRIM24 with Gifitinib. In the Western blot outcome: The expression of p-BAD and BCL-2 is reduced with tranfection of ShTRIM24, and decreased obviously with tranfection of ShTRIM24 and Gifitinib in A549 cell, total AKT have no change in the cells.

### 2.3 TRIM24在肺癌细胞系A549和PC9中对AKT信号通路的影响

在肺癌细胞系A549中干扰内源性TRIM24的表达引起AKT活性降低，p-AKT表达降低，而干扰TRIM24同时加入吉非替尼后AKT活性明显降低，p-AKT表达明显下降，而总的AKT没有明显的变化(图 3)。

**3:**
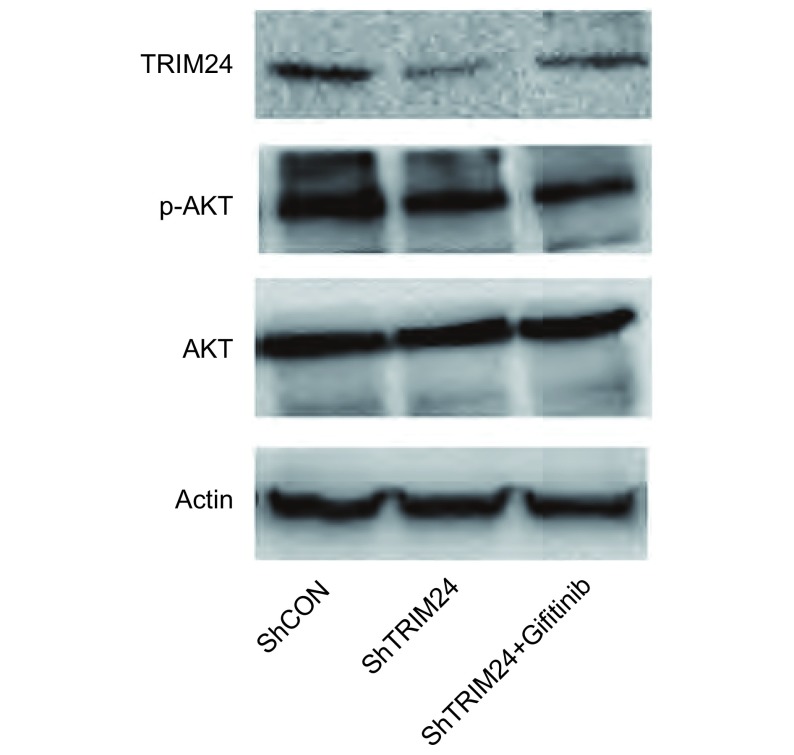
敲除TRIM24及同时加入吉非替尼引起AKT信号通路活性的变化。Western blot结果显示：敲除肺癌A549细胞中内源性TRIM24后p-AKT表达下调，而干扰TRIM24同时加入吉非替尼后p-AKT下降更为明显，总的AKT没有明显变化。 The change of AKT signal pathway with tranfection of ShTRIM24 and ShTRIM24 with Gifitinib. In the In the Western blot outcome: The expression of p-AKT is reduced with tranfection of ShTRIM24, and decreased obviously with tranfection of ShTRIM24 and Gifitinib in A549 cell, total AKT have no change in the cells.

### 2.4 TRIM24在肺癌细胞系A549和PC9中对AKT信号通路相关因子的影响

应用实时定量PCR和Western blot方法检测在肺癌细胞系中TRIM24对AKT信号通路相关蛋白的影响，结果显示：在A549细胞中干扰内源性TRIM24后引起AKT信号通路蛋白PIK3CA的mRNA及蛋白水平均下降，而干扰TRIM24同时加入吉非替尼后PIK3CA的mRNA及蛋白水平均下降更为明显，而P85和PTEN没有明显变化(图 4)。

**4:**
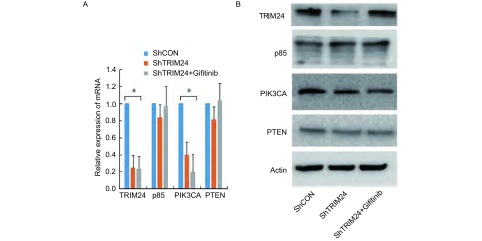
敲除TRIM24及同时加入吉非替尼引起AKT信号通路关键因子的变化。A：mRNA表达改变；B：蛋白表达。敲除肺癌A549细胞中内源性TRIM24后AKT信号通路相关蛋白PIK3CA的mRNA和蛋白均表达下调，而干扰TRIM24同时加入吉非替尼后PIK3CA的mRNA和蛋白表达降低更为明显，相关因子p85和PTEN未见明显变化。^*^：*P* < 0.05。 The change of protein related AKT signal path way with tranfection of ShTRIM24 and ShTRIM24 with Gifitinib. A: the expression of mRNA; B: the expression of protein. The expression of PIK3CA is reduce in mRNA and protein with tranfection of ShTRIM24, and decreased obviously with tranfection of ShTRIM24 and Gifitinib in A549 cell, the p85 and PTEN have no change. ^*^: *P* < 0.05.

## 3 讨论

以往研究^[12]^报道TRIM24 mRNA水平和蛋白水平在乳腺癌中过表达，且与雌激素受体(estrogen receptor, ER)和孕激素受体(progesterone receptor, PR)密切相关，是乳腺癌的一个不良预后指标。但TRIM24是如何促进肺癌发生发展这一分子机制尚不清楚，TRIM24是否与临床肺癌化疗耐药相关更未见报道。我们的前期研究结果^[15]^证实肺癌中的研究证实TRIM24的表达与肺癌的p-TNM分期和分化程度密切相关，且与细胞增殖因子Ki-67和周期相关蛋白CyclinD1、p-Rb密切相关，这一切表明TRIM24在肺癌发生发展中发挥着重要的作用。之前研究报道在乳腺癌细胞中TRIM24能够促进P53泛素化，从而负性调控P53蛋白的含量^[12]^，我们前期的研究结果证实在肺癌细胞中干扰内源性TRIM24后G_1_期细胞增多，而S期和G_2_/M期细胞减少，即干扰内源性TRIM24后阻滞细胞周期由G_1_期向S期转化，同时促进了细胞凋亡^[15]^。同时这次实验发现在吉非替尼作用下，在A549细胞中使用ShRNA干扰TRIM24可以提高吉非替尼对肿瘤增殖能力的抑制率，增加吉非替尼诱导细胞凋亡水平，同时引起凋亡相关蛋白的变化。上述结果强烈提示我们：TRIM24过表达能够影响肺癌细胞凋亡及相关基因的变化，能够影响肺癌细胞产生对EGFR-TKI的耐药性，但是TRIM24是如何导致凋亡变化，以及TRIM24又是如何介导肺癌细胞产生吉非替尼耐药的分子机制等，目前尚不清楚。近期有许多的研究报道^[16-18]^指出：EGFR非依赖性的PI3K-AKT通路持续激活在EGFR-TKI的耐药中发挥了重要作用，AKT信号通路可以通过多种途径抑制凋亡促进细胞存活。因此我们应用实时定量PCR及Western blot方法检测了AKT的活性及AKT信号通路相关蛋白的表达，研究发现在A549细胞中干扰内源性TRIM24的表达引起AKT活性及通路相关蛋白PIK3CA降低，而干扰TRIM24同时加入吉非替尼后AKT活性及通路相关蛋白PIK3CA降低更为明显，因此TRIM24可能通过与PIK3CA结合激活AKT信号通路，从而调控肺癌细胞对吉非替尼的耐药，更详细具体的分子机制我们将进一步进行探讨，这将为肺癌临床治疗提供重要的理论依据。
